# Exogenous melatonin reduces the inhibitory effect of osmotic stress on antioxidant properties and cell ultrastructure at germination stage of soybean

**DOI:** 10.1371/journal.pone.0243537

**Published:** 2020-12-15

**Authors:** Mingcong Zhang, Songyu He, Bin Qin, Xijun Jin, Mengxue Wang, Chunyuan Ren, Liang Cao, Yuxian Zhang

**Affiliations:** College of Agronomy, Heilongjiang Bayi Agricultural University, Daqing, People’s Republic of China; University of Agriculture, PAKISTAN

## Abstract

Understanding the relationship between exogenous melatonin and water deficit stress is crucial for alleviating the effects of water deficit stress at germination stage of soybean (*Glycine max (L*.*) Merrill*) in agriculture. This study investigated the effects of exogenous melatonin on soybean antioxidant properties and cell ultrastructure under water deficit stress induced by polyethylene glycol (PEG) 6000. The drought-sensitive soybean variety Suinong 26 was used as the material to study the effects of different concentrations of melatonin (0, 300, 500 μmol·L^-1^) soaking soybean seeds under drought stress (PEG-6000: 3% and 6%). The results showed that the germination rate (GR), germination potential (GP), germination index (GI) and radicle shape of soybean were affected negatively to different degrees under PEG stress. Moreover, stress induced by different PEG concentrations overproduced the content of reactive oxygen species (H_2_O_2_, O_2_^·−^) in cells, leading to increased lipid membrane peroxidation as electrolyte leakage (EL) and malondialdehyde (MDA) content, which resulted in impaired cell integrity. However, after seeds soaking with melatonin, the lipid peroxidation of the cell membrane was reduced, and the activities of antioxidant enzymes such as superoxide dismutase (SOD), peroxidase (POD), catalase (CAT), and ascorbate peroxidase (APX) further increased to minimize the excessive generation of ROS. Similar results were obtained for soluble protein and proline, that may help in regulating the osmotic pressure and maintain cellular integrity. With the interaction of these enzymes, compared with 300 μmol·L^-1^ melatonin, 500 μmol·L^-1^ melatonin could more effective to remove the ROS and reduce cell peroxidation. Overall, 500 μmol·L^-1^ melatonin performed better than 300 μmol·L^-1^. In conclusion, the seed soaking with melatonin promoted the germination of soybean seeds under water stress.

## Introduction

In recent past, drought has severely damaged the agricultural production more than any other environmental factor [1]. Arid environment severely restricts the production and development of agriculture. In arid or semi-arid environments, soil moisture deficiency during sowing stages affects seed germination, resulting in uneven seedling emergence, further decreased the number of plants and the yields of crop in the field [2]. Drought tolerance during seed germination is an important characteristic to ensure normal emergence and the planting density. Changes in the germination rate (GR), germination potential (GP), germination index (GI) and radicle shape can be used as indexes for the evaluation of seed drought tolerance during germination [3].

It is difficult to cultivate experimental plants under water stress by controlling watering to reduce soil moisture in the field production [4]. In our experiment, we used indoor culture methods in order to simulate the field crop germination experiments. PEG 6000 is an inert substance that can’t enter the cell wall, and it had been certified that plants were hard to absorb PEG with molecular weight exceeding 3000. According to the reported, PEG 6000 causes significant water stress to plant growth without any toxic effects [5]. Wherefore, PEG 6000 was used to simulate water stress in agricultural production [6]. In our preliminary experiment, we indicated that PEG 6000 concentration of 3% and 6% (w/v) had the effect of water deficit stress on Suinong 26, which is the main variety of soybean in Heilongjiang Province, China. The GP, GI and radicle length of seeds gradually decrease with increasing concentrations of polyethylene glycol (PEG), thus affecting the germination of seeds [7]. Furthermore, seed germination and vigor decreased as the number of stress degree days increased [8]. There are some other reports that inhibition of germination at the same water potential of NaCl and PEG resulted from osmotic effects rather than salt toxicity [9]. Therefore, people use various methods to improve the germination of seeds, like the phytohormone regulation of plant lateral root development [10] and regulation of drought-tolerant genes expression in rice [11] and soybean [12].

In water stress, the balance of reactive oxygen species (ROS) in plants is impaired, leading to the extra generation of ROS at a rate greater than the scavenging rate, and a large number of ROS especially superoxide anions (O_2_^·−^) and hydrogen peroxide (H_2_O_2_) constantly invade plant cells, thus the inhibition of plant growth [13]. In addition, with the increase in the degree of drought, the accumulation of ROS continuously destroys the stability of the membrane system, leading to the enhancement of lipid membrane peroxidation. The membrane permeability increases, so the electrolyte permeability also increases [14]. In this situation, the content of malondialdehyde (MDA) also increases [15] and further damages plant cells.

Plants can use self-regulation to adapt to adverse changes in the external environment. Antioxidant enzyme systems based on superoxide dismutase (SOD), peroxidase (POD), catalase (CAT) and ascorbate peroxidase (APX) can regulate the balance of oxidation in plants, and reduce the damage of ROS to plants. Proline and soluble protein are two osmosis regulating substances in plants that play an important regulatory role in drought and other stresses. These proteins can maintain the osmotic balance of cells, reduce the loss of water and remit the impact of adversity [3]. Proline has a strong hydration ability, which can protect proteins from degeneration and dehydration under osmotic stress, so as to maintain the water-retention capacity of cells [16]. In addition, proline is a protectant of many enzymes and thus protects the stability of the enzyme system [17]. With the increase in the degree of drought, proline and soluble protein in plants were accumulated continuously to cope with the impact of drought on hot pepper [18], soybean [19] and upland rice [20].

Melatonin (N-acetyl-5-methoxytryptamine), is an important indole that is widely distributed in plants and animals [21] and was initially identified in the bovine pineal gland by Lerner [22]. With the advancement of research, it was found that melatonin can regulate the growth of plants [23]. It acts as antioxidant [24] that can directly remove superoxide free radicals (O_2_^·−^) and H_2_O_2_ [25, 26], improves the antioxidant enzyme activity of plants under drought stress [3], promotes the germination and lateral root formation of seeds under adverse stress conditions [27].

At present, melatonin has been extensively reported in maize [28], wheat [29] and rice [30]. However, studies on alleviating water stress during the germination of soybean seeds have rarely been reported. PEG is a neutral permeable active polymer with a specific molecular weight. It is the most used substance in plant water shortage research because of the resulting reduction in the water potential after PEG use induces dehydration [31]. Therefore, in this study, Suinong 26 was used as an experimental material soaked with melatonin in advance. Seed germination, radicle shape, lipid peroxidation products, antioxidant enzyme activity and osmosis regulating substances under PEG-6000 induced water stress were determined. Therefore, the purpose of this study seeks to explore the effects of the seed soaking with melatonin on alleviating drought of soybean seeds germination, it could provide a theoretical basis for improving drought resistance of soybean seedlings in actual production.

## Materials and methods

### Experimental variety

In this experiment, Suinong 26 (Glycine max (L.) Merr.), which has an unlimited pod bearing habit and a growth period of approximately 120 d, was used as the experimental material. Melatonin (C_13_H_16_N_2_O_2_) was purchased from Sigma Aldrich and had a relative molecular weight of 232.28 with a purity of > 99%.

### Seed treatment and germination

**Fig 1:**
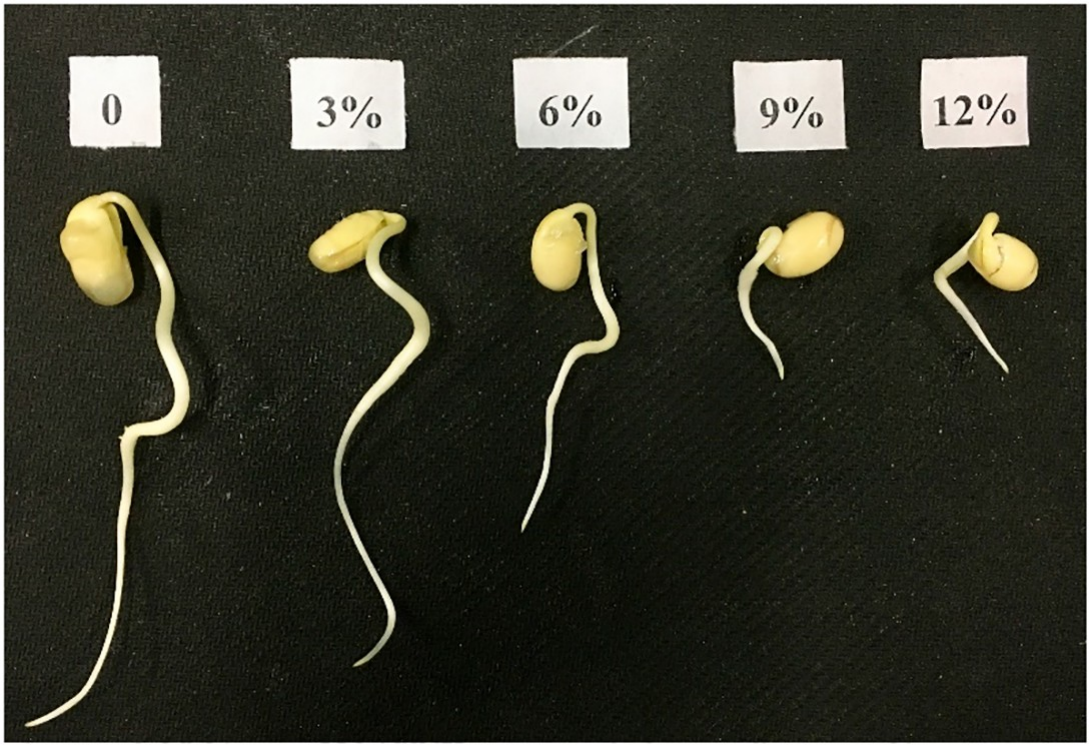
Germination of seeds with different concentrations of PEG. 0: deionized water, no water stress; 3%, 6%, 9% and 12% were indicated 3%, 6%, 9% and 12% PEG6000 (w/v).

The experiment was carried out in the agronomy laboratory of Heilongjiang Bayi Agricultural University in Daqing city, Heilongjiang Province. On the basis of preliminary experimental results (Fig 1), the PEG 6000 (W/V) concentrations used in this experiment were 0 (deionized water: no water stress), 3% and 6%, and the corresponding water potentials were 0MPa, -0.02MPa and -0.07 MPa [32]. The treatment concentrations of melatonin were M1:300 μmol·L^-1^ and M2: 500 μmol·L^-1^ (An amount of 0.232g of melatonin was accurately weighed. Afterward, the melatonin was dissolved in an appropriate amount of anhydrous ethanol, and deionized water was used to bring the volume to 100ml. The prepared solution represented a mother liquor of 10,000 μmol·L^-1^ and was stored at 4°C. During the test, the corresponding volume of mother liquor was diluted to the required concentration according to the test method). Deionized water was used in place of melatonin as the control (CK). Therefore, a total of 9 treatments were established: 0-CK, 0-M1, 0-M2, 3%-CK, 3%-M1, 3%-M2, 6%-CK, 6%-M1, and 6%-M2.

Soybean seeds were selected with clean, healthy, undamaged, and exhibited full granules, disinfected in 8% sodium hypochlorite solution for 5 min, rinsed with distilled water several times and then dried. The seeds were subsequently immersed in equal amounts of deionized water (CK), 300 μmol·L^-1^ melatonin (M1) or 500 μmol·L^-1^ melatonin (M2) under dark conditions for 12 h. Afterward, the soybean seeds were transferred into a 12 cm × 12 cm sterile culture dish that contained 2 pieces of filter paper, approximately 30 seeds (according to the size of culture dish) were evenly placed in each culture dish, and each treatment was repeated 3 times. The solutions used for germination included deionized water, 3% PEG and 6% PEG. The germination test was performed in a constant-temperature incubator with a day/night cycle of 25°C /18°C. The filter paper and the evaporated germination solutions were replaced and replenished, respectively, at 12:00 p.m. every day. Germination was observed and recorded daily. After 7 d, the relevant indexes were measured

## Determination of indexes and methods

### Germination parameters

Germinationpotential(%)=(numberofseedsgerminatedin3d/totalnumberofseeds)×100%

Germinationrate(%)=(numberofseedsgerminatedin7d/totalnumberofseeds)×100%

$$\text{Germination}\;\text{index}\;(\%)\;=\;\sum ({\text{G}}_{\text{t}}/{\text{D}}_{\text{t}}),$$

where G_t_ represents the number of germinated seeds on day t and D_t_ represents the number of germination days [33].

### Morphogenesis index

At 7 d after germination, ten radicles were randomly selected in each treatment. The radicles were scanned by an Epson root scanner, and the shape of the radicals was subsequently determined. The length, surface area, volume and diameter of the radicle were analyzed by WinRHIZO root analysis software. Moreover, the fresh weight and dry weight of the radicles were measured.

### Measurement of osmoregulatory substances

The enzyme (SOD, POD, CAT, and APX) mixtures consisted of 5 mL of ice-cold phosphate buffer (0.05 M pH 7.8), 1% (w/v) polyvinylpyrrolidone (PVP) and 2 mM EDTA. Radicle tip tissues (0.5 g each) were ground, centrifuged at 12,000 g for 20 min at 4°C, and the supernatant was used to measure the antioxidant enzyme activity.

#### SOD

The assay mixture consisted of 50 mM sodium carbonate bicarbonate buffer (pH 9.8) that consisted of 0.1 mM EDTA, 0.6 mM epinephrine and the enzyme at a total volume of 3 ml. The adrenochrome formation at 4 min after the addition was recorded by a UV–Vis spectrophotometer at 470 nm. One unit of SOD activity was defined as the amount of enzyme required to cause 50% inhibition of epinephrine oxidation under the experimental conditions (modified method of Misra [34]).

#### CAT

The assay mixture consisted of 1000 μL of 100 mM KH_2_PO_4_ buffer (pH 7.0), 400 μL of 200 mM H_2_O_2_ and 100 μL of the enzyme at a total volume of 1.5 mL. The decrease in H_2_O_2_ was monitored at 240 nm (modified method of Beers [35]).

#### POD

The assay mixture consisted of 40 mM NaH_2_PO_4_ buffer (pH 6.1), 2 mM H_2_O_2_, 9 mM guaiacol and 50 μL of enzyme extract at a total volume of 5 mL. The increase in absorbance at 420 nm was measured by a UV–Vis spectrophotometer at 30 s intervals for a maximum of 2 min. Enzyme specific activity was presented as the number of micromoles of H_2_O_2_ reduced per minute per milligram of protein (modified method of Egley [36]).

#### APX

The enzyme activity of APX was assayed according to the method of Nakano [37]. The reaction mixture for the POD consisted of 50 mM potassium phosphate, pH 7.0, 5 mM ascorbate, 0.1 mM H_2_O_2_ and 0.1 mM EDTA at a total volume of 2 ml. The reaction was started by the addition of the 1.7ml EDTA (0.1 mM), 0.1ml ascorbate (5 mM) and 0.1ml H_2_O_2_ (20 mM). The absorbance change at 30 s after the addition was recorded by a UV–Vis spectrophotometer at 290 nm (4 replications).

The total antioxidant enzyme activity was expressed as enzyme units of activity per gram of fresh weight (U/g FW).

### Measurement of lipid peroxidation (MDA) and Electrolyte Leakage (EL)

Approximately 0.2 g of fresh radicle tip tissue was ground in 0.25% 2-thiobarbituric acid (TBA) in 10% trichloroacetic acid (TCA) via a mortar and pestle, and the MDA was measured by Heath and Packer [38]. The electrolyte leakage (EL) was measured according to the methods of Sutinen [39].

### Measurement of osmoregulatory substances (soluble protein and proline)

Approximately 0.1 g of fresh radicle tips were taken from each treatment and then ground in a mortar with 1 mL of phosphate-buffered solution (PBS, pH = 7.8). The soluble protein content was measured according to the methods of Zhang [27].

The free proline content was determined according to the method of Bates [40]. Approximately 0.5 g of fresh radicle tip samples from each group were homogenized in 3% (w/v) sulfosalicylic acid, after which the homogenate was filtered through filter paper. After the addition of acid ninhydrin and glacial acetic acid, the resulting mixture was heated at 100°C for 1 h in a water bath. The reaction was then stopped by via an ice bath. The mixture was then extracted with toluene, and the absorbance of the fraction with toluene aspired from the liquid phase was read at 520 nm. The proline concentration was determined via a calibration curve and was expressed as μmol g^−1^ FW.

### Hydrogen peroxide (H_2_O_2_) concentration

The H_2_O_2_ content in the radicle tip samples was colorimetrically measured as described by Mukherjee and Choudhuri [41]. The radicle tip samples were extracted with cold acetone to measure the H_2_O_2_ levels. An aliquot (1 mL) of the extract solution was mixed with 200 μL of 0.1% titanium dioxide in 20% (v/v) H_2_SO_4_, and the mixture was then centrifuged at 6000 g for 15 min. The intensity of the yellow color of the supernatant was measured at 415 nm.

### Superoxide anion (O_2_^·−^) measurements

Approximately 0.2 g of fresh radicle tips were homogenized under a cold (-196°C) N_2_ atmosphere in 100 mM sodium–phosphate buffer (pH 7.2) containing 1 mM diethyl dithiocarbamate to inhibit SOD activity. O_2_^·−^ was then measured according to the methods of Chaitanya and Naithani [42], and O_2_^·−^ formation was expressed as the change in absorbance (Δ*A*) at 540 nm per minute per milligram of protein.

### H_2_O_2_ and O_2_^·−^ fluorescence staining

The fluorescence staining methodology mainly refer to the methods of Sandalio [43]. Root tips with different treatments were cut to the length of approximately 10 mm and placed in black shaded with 5 mL centrifuge tube, dihydroethidium (DHE) staining solutions and 2’,7’-dichlorodihydrofluorescein diacetate (DCF-DA) were added (For O_2_^·−^: 5 μL of DHE mother liquor was dissolved in 20 mL of DMSO; For H_2_O_2_: 25μL DCF-DA mother liquor was dissolved in 10 mL DMSO). After they had stained for approximately 10 min, the cells were washed 3 times with 10 mmol·L^-1^ Tris-HCl and imaged under an Olympus SZX16 fluorescence microscope. The degree of membrane lipid damage was determined according to the methods of Yamamoto [44].

### Ultrastructural observations of apical tissue

The ultrastructure of the apical tissue was observed via a transmission electron microscope (JEM-2100Plus, Japan). Fresh radicle tip samples were fixed in 4% glutaraldehyde for 6 h. Afterward, the samples were rinsed with phosphate buffer fixated with 0.1 M osmic acid and rinsed again, after which the tissue samples dehydrated with ethyl alcohol. The samples were subsequently analyzed by transmission electron microscopy (TEM) [45].

### Statistical analysis and drawing

Excel 2019 was used for data processing and tabulation, SPSS 17.0 software was used to for one-way analysis of variance (ANOVA), and the mean differences were compared by the least significant difference (LSD) test. Comparisons in which *P*<0.05 were considered significantly different. Origin 2018 software was used for the construction of all figures, and the spread of values is shown as error bars representing the standard error of the means.

## Results

### Effects of exogenous melatonin on the germination parameters of soybean under PEG stress

In our study, compared with no water stress, the germination rate, germination potential and germination index of soybean seeds decreased under 3% and 6% PEG stress. In addition, the higher the degree of decrease as the increase of water stress. PEG stress significantly inhibited the normal germination of soybean seeds. However, when soaked with melatonin, the germination rate, germination potential and germination index of soybean seeds increased significantly under the stress of 3% PEG. Compared with 3%-CK, the germination rate, Germination potential and germination index with 3%-M1 and 3%-M2 increased by 27.3% and 31.8%, 20.8% and 20.8%, 23.9% and 26.1% (*P*<0.05), respectively. A similar phenomenon was found under 6% PEG stress. In addition, according to the coefficient of variation of the germination parameters, the germination rate varied more than the germination potential and the germination index.

### Effects of exogenous melatonin on radicle shape and biomass of soybean under PEG stress

To a certain extent, water deficit affects the growth of plant roots [46]. Wu [47] showed that water stress caused by PEG significantly inhibited the radicle length of three leguminous species. It can be seen from the data in Table 2, compared with the absence of PEG stress, M1 and M2 significantly increased the radicle length, surface area and volume of soybean seeds. Moreover, compared with that of M1, the treatment with M2 resulted in better performance. However, in the absence of seed soaking in melatonin, PEG stress reduced the radicle length, surface area, volume and diameter of the soybean radicles, and the reductions under 6% PEG were more severe than those under 3% PEG. The radicle length, surface area, volume, and diameter of soybean, compared with the control under water stress caused by PEG (3%-CK and 6%-CK), increased significantly after melatonin treatment (M1 and M2). These findings indicated that melatonin could alleviate drought stress during the germination of soybean seeds. Moreover, the effects of melatonin application were more obvious under 3% PEG.

In terms of radicle morphology and biomass, the dry weight and fresh weight of the radicles were sensitive with water stress, and the coefficients of variation were 19.00% and 18.63%, respectively. The root length and root surface area were relatively stable, with coefficients of variation 6.49% and 7.54%, respectively.

### Effects of soaking with melatonin on the H_2_O_2_ and O_2_^·−^ of soybean radicles under PEG stress

**Fig 2:**
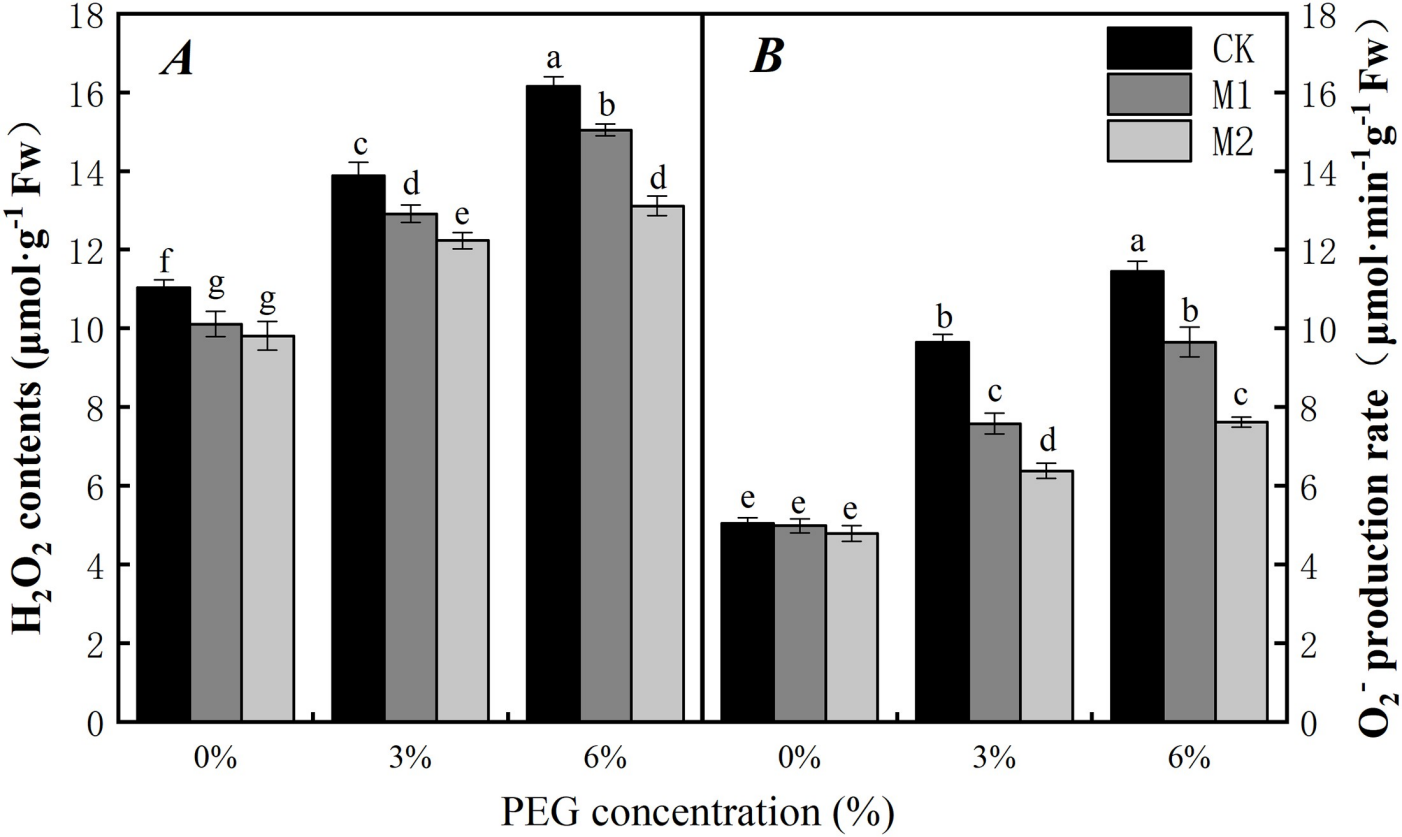
Effect of soaking with melatonin on H_2_O_2_ (A) contents and O2·− (B) production rate of soybean radicle under PEG stress. 0%, 3% and 6% were indicated 0%, 3% and 6% PEG6000 (*w/v*). Values are the means of three replicates ± S.E. Bars with different letters are significantly different at *P*≤0.05.

Water stress can cause the peroxidation of plant cells and the accumulation of excess ROS, which can cause toxic effects on cells. In the absence of water stress, the application of melatonin can significantly reduce the content of H_2_O_2_ in soybean radicles (Fig 2A). H_2_O_2_ contents in the M1 and M2 treatments decreased by 8.87% and 11.83%, respectively, when compared with that of the control (0-CK), but there was no significant difference in the rate of O_2_^·−^ production (Fig 2B).

Compared with no PEG6000 stress, PEG6000 stress can significantly increase the content of H_2_O_2_ and the production rate of O_2_^·−^ in soybean radicles in this study. Under 3% and 6% PEG stress, the content of H_2_O_2_ in the soybean radicles increased by 24.19% and 44.44% respectively (*P*<0.05), and the production rate of O_2_^·−^ increased by 48.47% and 131.29% respectively (*P*<0.05). Moreover, water stress induced the accumulation of H_2_O_2_ and O_2_^·−^ in the soybean radicles.

After soaking with melatonin, the content of H_2_O_2_ and the production rate of O_2_^·−^ in soybean radicles under PEG stress were significantly decreased. Under 3% PEG stress, compared with the CK treatment, the percent reduction in response to the M1 and M2 treatments was approximately 6.06% and 12.27% for H_2_O_2_ and 21.60% and 31.83% for O_2_^·−^, respectively. The same pattern of reduction was approximately 7.38%, 18.36% and 15.21%, 33.60% (*P*<0.05) for 6% PEG when compared with the control values. Exogenous melatonin could control the accumulation of H_2_O_2_ and O_2_^·−^ and thus reduce the degree of soybean membrane lipid damage.

### The histochemical staining and transmission electron micrographs of root tips revealed the effect of soaking with melatonin under PEG stress

**Fig 3:**
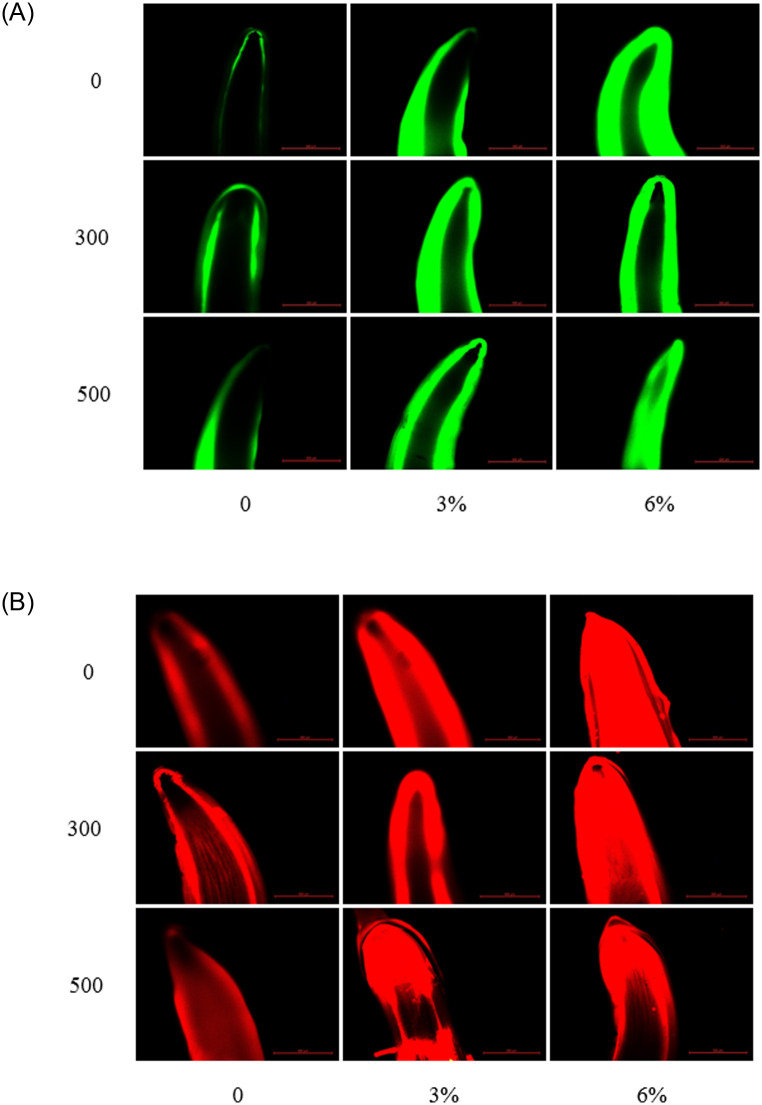
H_2_O_2_ and O2·− staining of soybean radicle root tips under different treatments. H_2_O_2_ on the left and O_2_^·−^ on the right. The horizontal axis is PEG concentration, the vertical axis is melatonin concentration, and the scale length is 500 μm.

In accordance with the methods of Sandalio [43], H_2_O_2_ and O_2_^·−^ fluorescence staining was performed on the root tips in each treatment. As shown in Fig 3, with increasing PEG stress, the intensity of green (H_2_O_2_) and red (O_2_^·−^) fluorescence in the root tips of the radicle also increased. However, the fluorescence intensity decreased after the seeds were soaked with melatonin. These results indicated that the H_2_O_2_ content and O_2_^·−^ production rate of soybean radicles under PEG stress were increased. However, when the seeds were soaked with melatonin, the H_2_O_2_ content and O_2_^·−^ production rate were diminished, and the damage of soybean radicles was reduced. In addition, the different concentrations of melatonin had different alleviating effects on the radicles of soybean under different PEG stress.

**Fig 4:**
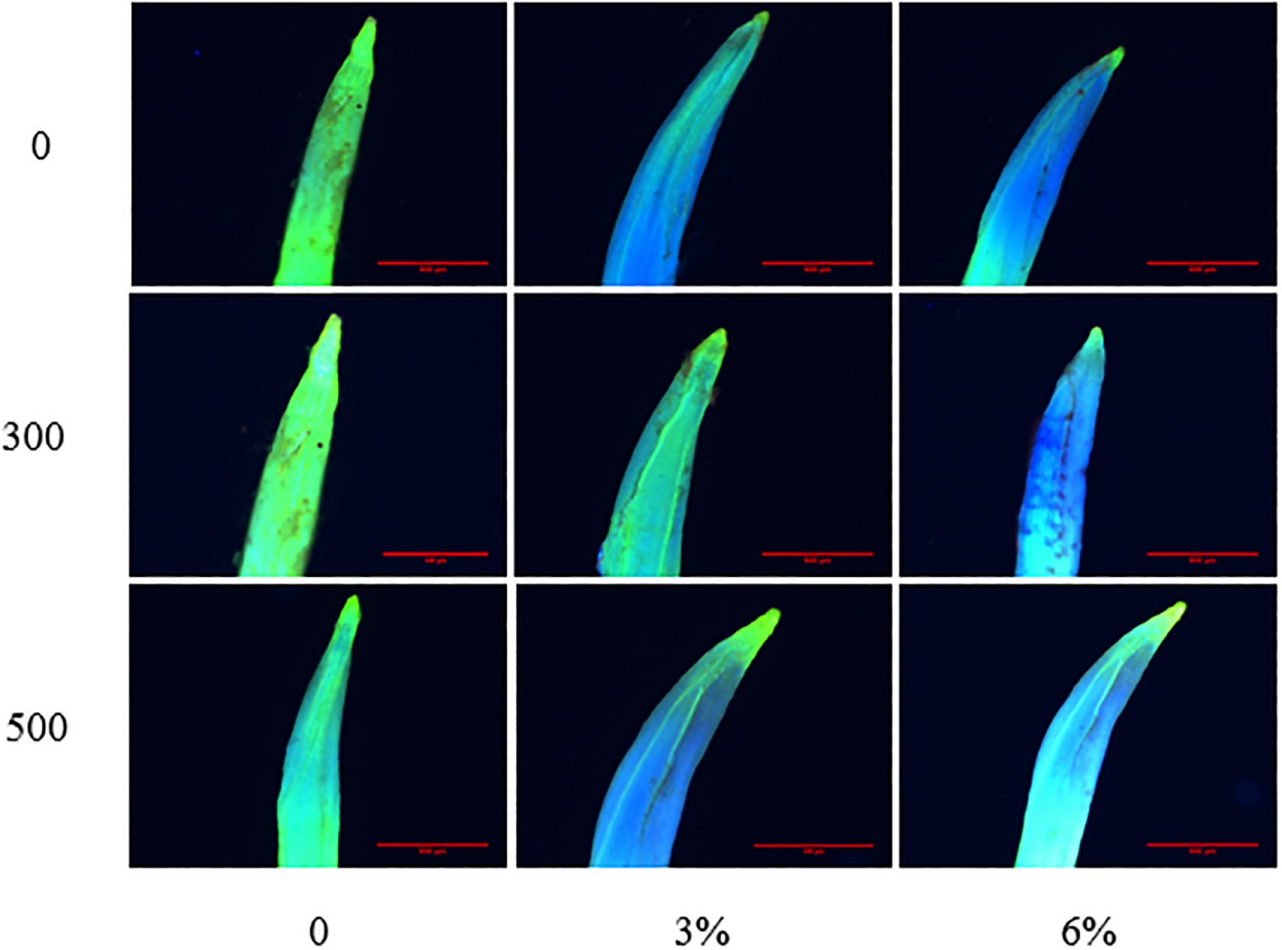
ROS staining of soybean radicle tips on different treatments. The horizontal axis is PEG concentration, the vertical axis is melatonin concentration, and the scale length is 500 μm.

The more blue areas were stained in plant root tips, the higher the amount of membrane lipid peroxidation and the greater the damage to cells. In our study, the blue areas of the root tips of the soybean radicles increased, indicating that the membrane lipid peroxidation of soybean increased under PEG stress (Fig 4). However, when the seeds were soaked with melatonin, the blue portion of the root tip decreased, indicating that melatonin could alleviate the effects of water stress on cells. However, the indicates that the melatonin treatment with different concentrations had different remission effects at different water stress levels.

**Fig 5:**
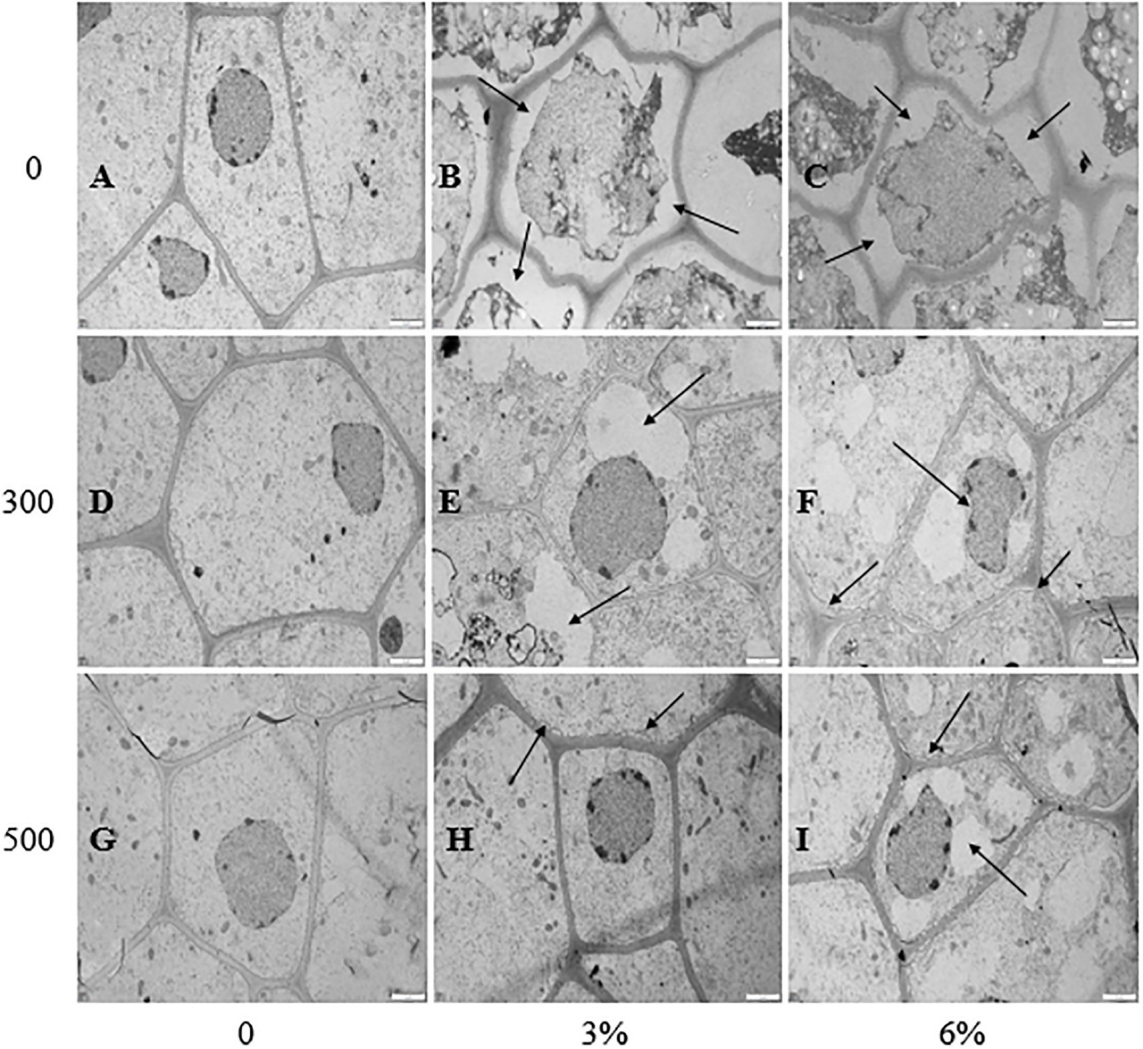
Transmission electron micrographs of ultrathin sections of cells from root tips treated with different treatments. The horizontal axis is PEG concentration, the vertical axis is melatonin concentration, the scale length is 2 μm, 2000 times magnified.

As shown in Fig 5, the root tip cells of soybean radicles developed well and free of damage in the absence of PEG stress (Fig 5A, 5D and 5G). When the seeds were subjected to PEG stress, the integrity of the cell was destroyed, resulting in incomplete cellular components. The main manifestations were plasma cell dissolution, nuclear deformation and severe damage of mitochondrial plastid network and other organelles (Fig 5B and 5C). However, the application of the soaking with melatonin alleviated the negative effects of PEG stress, decreased the degree of plasmolysis, and the nucleus and mitochondria remained intact, but the nucleus was still deformed and the cytoplasm was disordered (Fig 5E, 5F, 5H and 5I). In addition, the effects of 500 μmol·L^-1^ melatonin were better than that of 300 μmol·L^-1^.

### Effects of soaking with melatonin on the EL and MDA content in radicles of soybean under PEG stress

**Fig 6:**
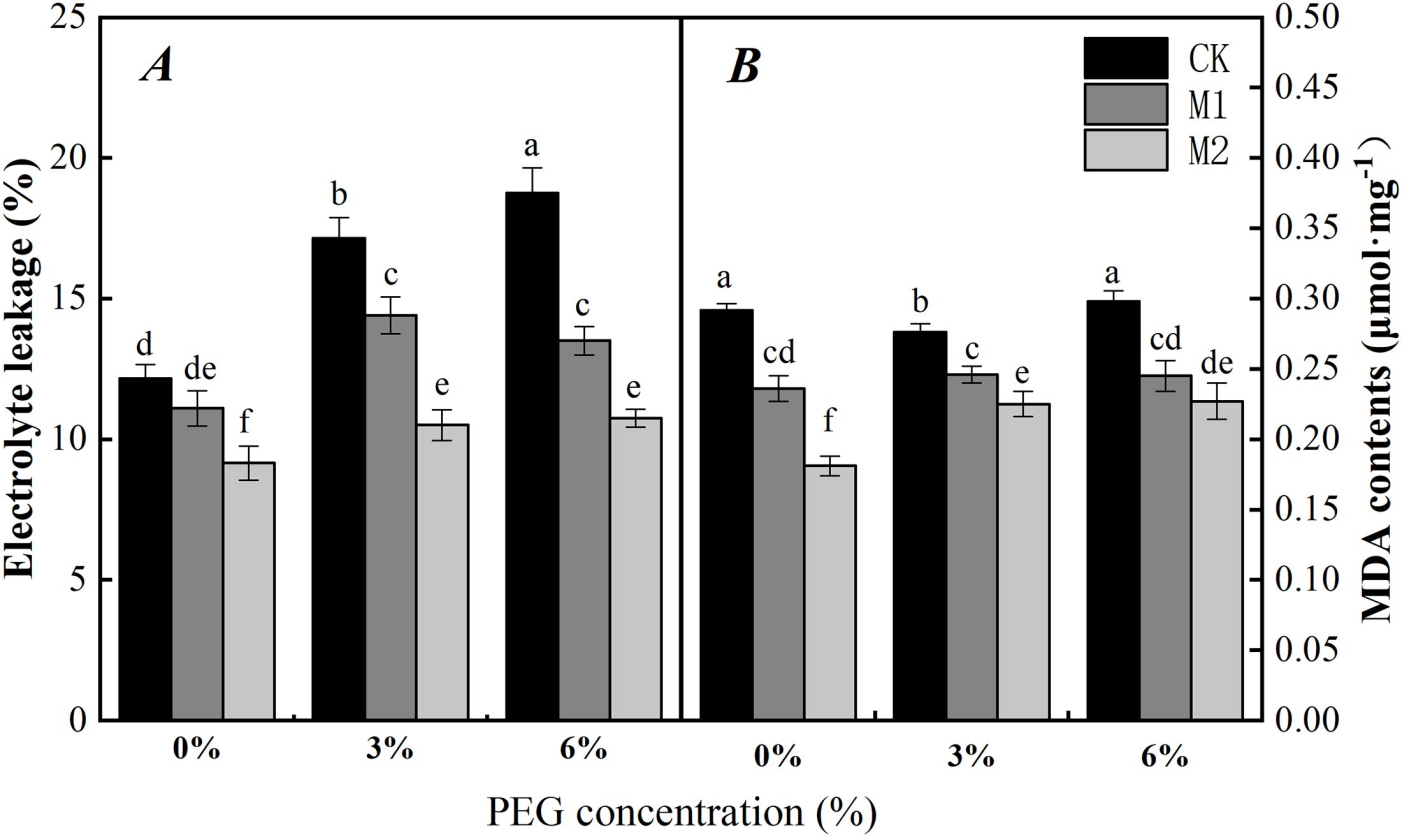
Effect of soaking with melatonin on EL (A) and MDA (B) of soybean radicle under PEG stress. 0%, 3% and 6% were indicated 0%, 3% and 6% PEG6000 (*w/v*). Values are the means of three replicates ± S.E. Bars with different letters are significantly different at *P*≤0.05.

The effects of adverse environmental conditions can cause membrane lipid peroxidation, which, along with its products, can disrupt the function and integrity of biofilms and even lead to cell death [48]. We can see from Fig 6 that melatonin treatment can significantly reduce the EL and MDA content in soybean radicles in the absence of PEG stress. However, in the absence of soaking with melatonin, the EL conductivity of soybean radicles was significantly increased under PEG stress when compared with no PEG stress. In addition, the EL conductivity of soybean radicles was increased by approximately 42.04% and 54.99% occurred in response to 3% and 6% PEG, respectively (Fig 6A).

Compared with those in the CK treatment, the MDA contents of soybean radicles in the M1 and M2 treatments significantly decreased by approximately 6.23% and 17.89%, respectively, and the EL decreased by approximately 15.84% and 38.89%, respectively, under 3% PEG. When the PEG concentration reached 6%, compared with those in the CK treatment, the MDA contents in the M1 and M2 treatments significantly decreased by 17.67% and 28.05%, respectively, and the EL decreased by approximately 28.35% and 42.71%, respectively (Fig 6A and 6B). The M2 treatment had a more significant effect on the EL of and MDA content in soybean radicles than did the M1 treatment under PEG stress.

### Effects of soaking with melatonin on the antioxidant enzyme activity and osmotic adjustment of soybean radicles under PEG stress

**Fig 7:**
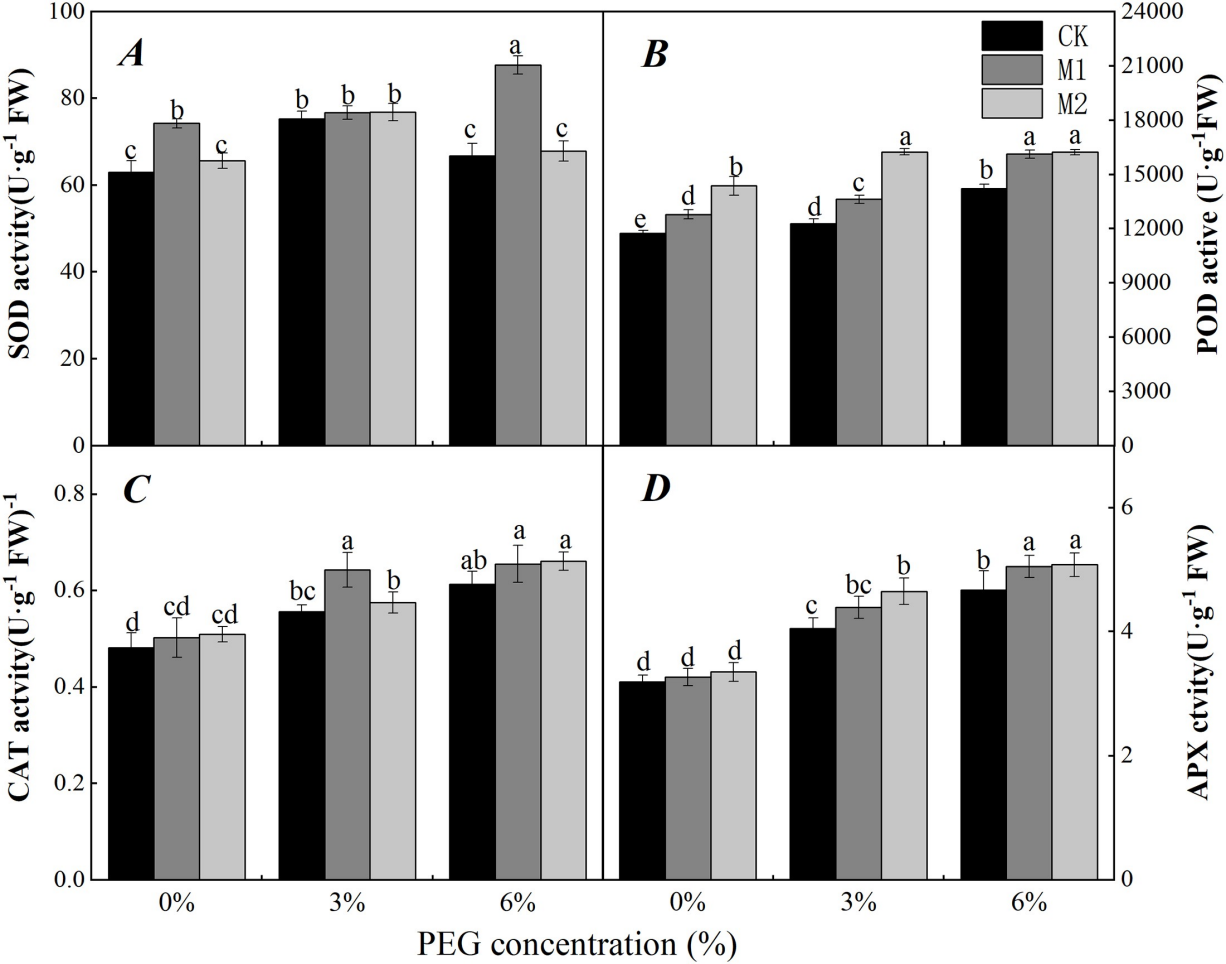
Effect of soaking with melatonin on antioxidant enzyme activity of soybean radicle under water stress caused by PEG. 0%, 3% and 6% were indicated 0%, 3% and 6% PEG6000 (*w/v*). Values are the means of three replicates ± S.E. Bars with different letters are significantly different at *P*≤0.05.

In this study, in the absence of soaking with melatonin, the antioxidant enzyme activity of soybean radicles increased by PEG when compared with that under no water stress (0-CK). The SOD activity first increased but then decreased with increasing PEG concentration (Fig 7A). The activity of POD, CAT and APX increased with increasing PEG concentration (Fig 7B–7D).

When the seeds were soaked with melatonin, the antioxidant enzyme activity of soybean radicles under water stress further increased. Under 3% PEG treatment, the SOD activity of soybean radicles was greater than that in the control, but the difference was not significant (Fig 7A). Compared with that in the CK treatment, the POD activity in the M1 and M2 treatments significantly increased by 10.14% and 31.48%, respectively (Fig 7B). In addition, the activity of CAT increased by 16.36% (*P*<0.05) following M1 treatment, and the APX activity increased by 13.72% (*P*<0.05) following M2 treatment (Fig 7C and 7D). When the PEG concentration reached 6%, the SOD activity was significantly increased by M1 treatment, while POD and APX activity increased by M1 and M2 treatments, respectively. Exogenous melatonin thus promotes a further increase in antioxidant enzyme activity to remove excess ROS under water stress.

**Fig 8:**
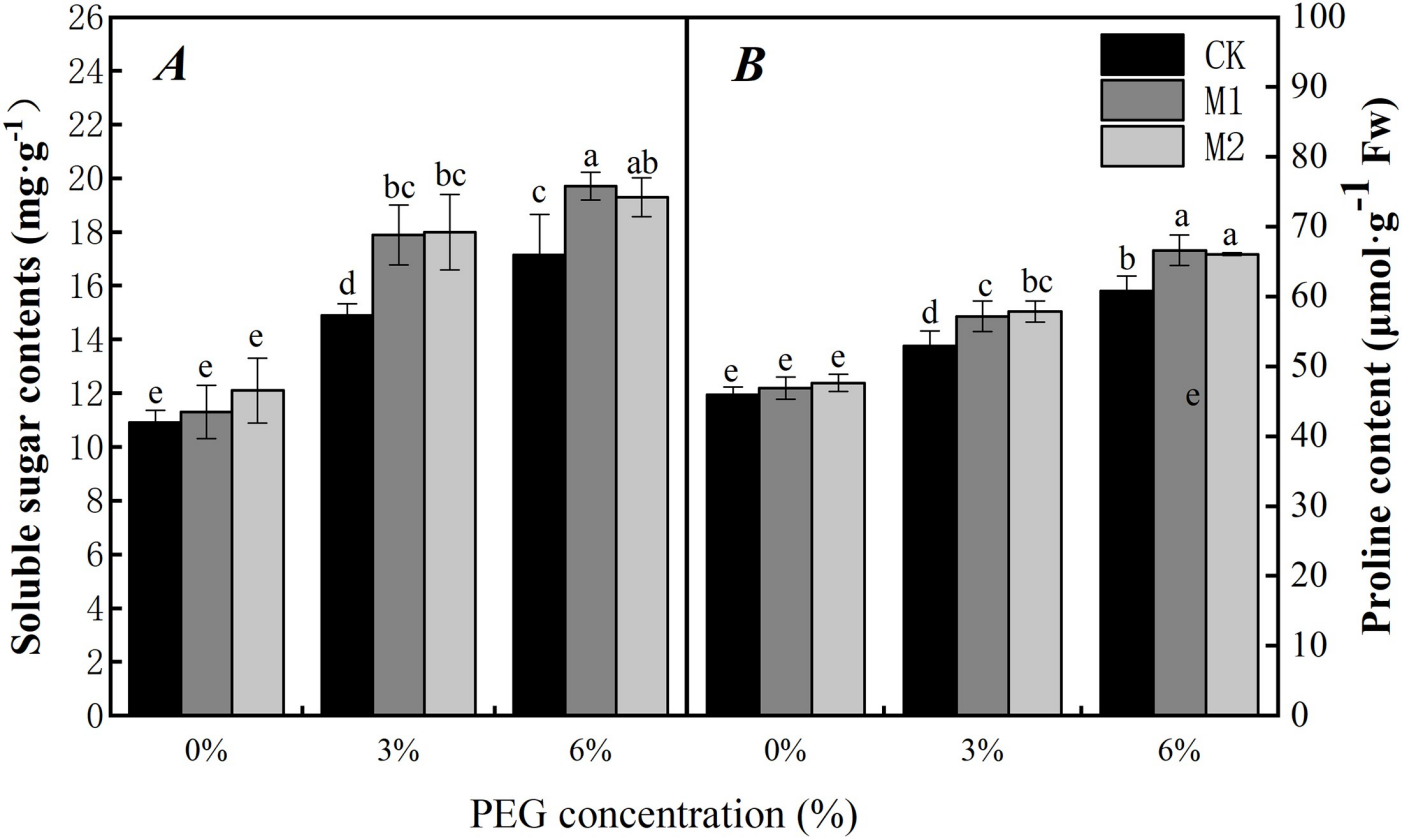
Effect of soaking with melatonin on soluble protein (A) and proline (B) of soybean radicle under PEG stress. 0%, 3% and 6% were indicated 0%, 3% and 6% PEG6000 (*w/v*). Values are the means of three replicates ± S.E. Bars with different letters are significantly different at *P*≤0.05.

The osmotic adjustment of plants under water stress is another part of the plant defense system [49]. Many studies have shown that both the soluble protein and proline contents increase in plants under water stress, thereby protecting cell integrity [50–52]. In our study, we found that the contents of soluble protein and proline within soybean radicles increased with increasing PEG concentration only under water-stress when compared with that under no water stress (Fig 8). The increase in these two indicators shows that PEG stress induces the osmotic defense system of soybean radicles and regulates cell water balance.

The treatments of melatonin soaking with 3% and 6% PEG stress, compared with the control treatments with 0% PEG stress, the contents of the soluble protein and proline in the soybean radicles were significantly increased. However, there was no significant difference between different melatonin treatments. In addition, we also found the proline content increased less than soluble protein at different PEG concentration, it may be due to the different effects of melatonin on the regulation of osmotic action.

### Principal component analysis

**Fig 9:**
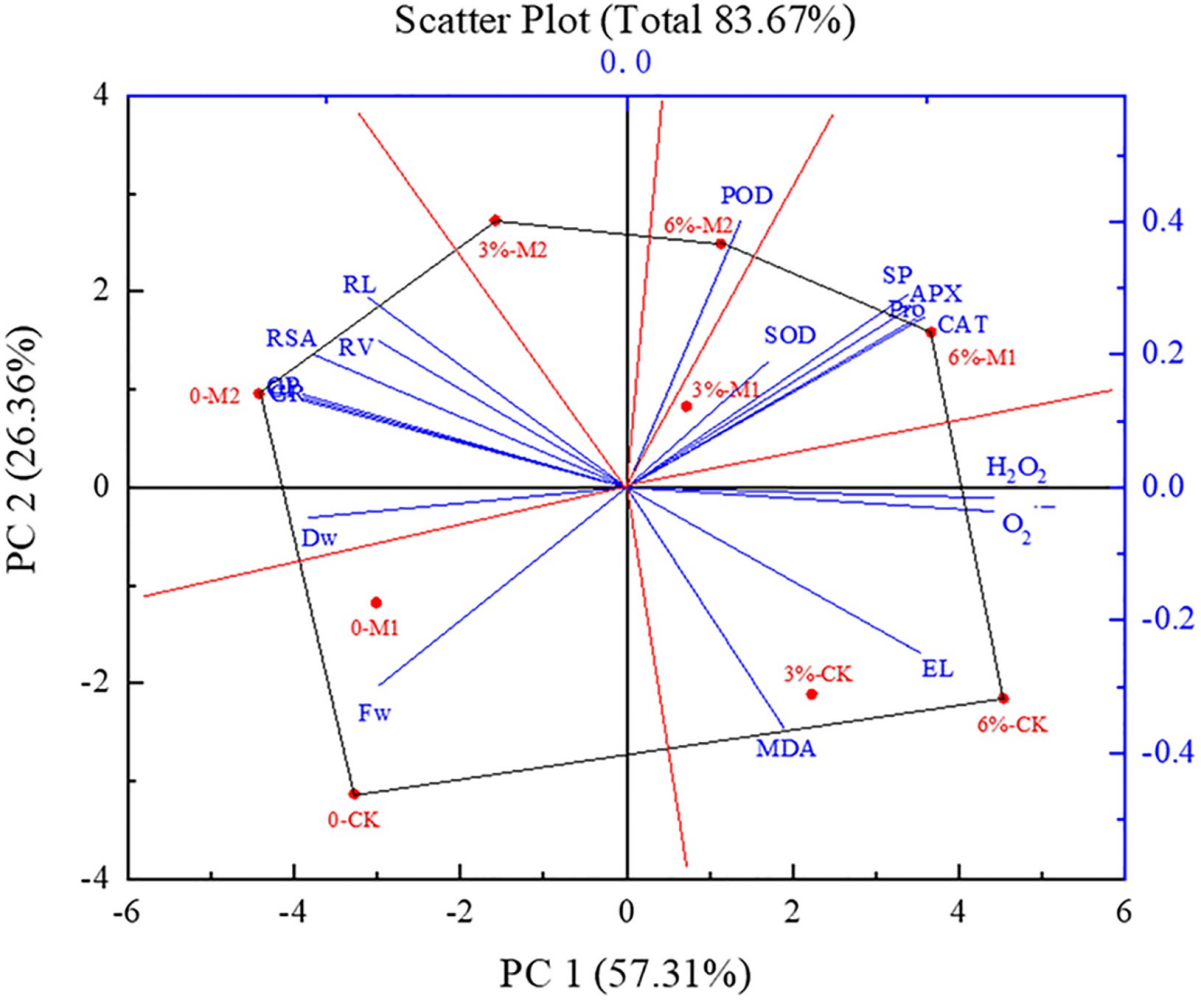
Analysis of all indicators of soybean seeds based on GGE biplot under different treatments.

The effects of the first and second principal components were PC1 = 57.31%, PC2 = 26.3%, respectively, and the genotype plus genotype × environment (GGE) biplot reflected 83.67% of all true information with each treatment (Fig 9). The analysis results were highly reliable. A polygon was formed by connecting the outermost treatment points (0-CK, 0-M2, 3%-M2, 6%-M2, 6%-M1, 6%-CK) with straight lines. The six red lines perpendicular to each side from the center point divide the entire figure into six areas. All indicators are distributed among the six regions. We can see that four indicators H_2_O_2_, O_2_^·−^, MDA and EL are divided into 6%-CK treatment areas. These indicators, therefore, have the greatest impact under the 6%-CK treatment, which is also reflected in the previous analysis. In addition, the angle between these four indicators was less than 90°, indicating a significant positive correlation between them. Moreover, the angle between H_2_O_2_ and O_2_^·−^ was relatively small, showing that the correlation between the two was relatively strong. Therefore, it can be preliminarily concluded that the amounts of H_2_O_2_, O_2_^·−^, MDA and EL in soybean radicles in the 6%-CK treatment were relatively high; thus, the degree of damage was greater than that in the other treatments.

SOD, APX, CAT, soluble proteins and proline were distributed in the region in which the 6%-M1 treatment was the apex, indicating that these indicators were highest under 6%-M1 treatment. There is a positive correlation between them because the angle is less than 90°. In addition, the angle between SOD, POD, APX, CAT, soluble proteins, proline, H_2_O_2_ and O_2_^·−^ is also less than 90°, indicating that the accumulation of ROS under PEG stress could induce the increase of antioxidant enzyme activity and osmotic adjustment substances. However, POD was an antioxidant enzyme which was reduced by 6%-M2 treatment. PEG stress led to an increase in the activity of antioxidant enzymes and osmosis-regulating substances, and the activity and content of the two substances further increased in response to melatonin soaking to remove excess H_2_O_2_ and O_2_^·−^, regulate cell osmotic potential, and alleviate cell damage, all of which are essentially consistent with previous findings (Figs 7 and 8). These results reflect the regulatory effect of melatonin on the antioxidant enzyme activity and osmosis-regulating contents under PEG stress. In addition, the angle between CAT and APX was relatively small, indicating that the synergy between scavenging ROS and free radicals is strong. This finding may be due to the way in which these scavengers act in the antioxidant system [53].

Other indicators such as root length, root surface area, root volume, dry weight, germination rate; germination potential and germination index were distributed within the 0-M2 region, while fresh weight was distributed within the 0-CK region. These results showed that, compared with the other treatments, the 0-M2 treatment had a significant effect on the germination parameters, morphological index and dry weight of soybean. The fresh weight was the greatest in the 0-CK treatment. Moreover, the angle between the root length, root surface area, and root volume was less than 90°, indicating a positive correlation; the germination parameters were also positively correlated, and the correlation was relatively strong.

## Discussion

Seed germination is a crucial stage at the beginning of the growing period [54]. Many adverse external factors, such as temperature, salinity and heavy metals in the environment, can affect the germination of seeds [55, 56]. However, water is also one of the most important factors, and seeds can maintain a good germination state only after reaching optimal levels of water absorption [57]. As a regulator of indoles in plants, melatonin can improve the germination rate of seeds under adverse stress conditions [58] and increases the number and length of lateral and adventitious roots [59]. In our study, the germination rate, germination potential and germination index, radicle dry weight and fresh weight of soybean seeds decreased significantly under PEG stress. The same results were found for the radicle length, surface area, and volume. Moreover, water stress induced by PEG could inhibit the germination of soybean seeds. These results were consistent with those of Mether [6] who used PEG-6000 to simulate water stress to study the germination characteristics and drought resistance of mungbean seeds. However, in the present study, after the seeds were soaked with melatonin, all of the above indicators showed that the drought stress effects were alleviated (Tables 1 and 2); and some similar results have also been reported for cucumber [3]. In addition, under different PEG concentrations, the effects of the different concentrations of melatonin on the soybean seeds differed. Soybean varieties with Sunnong 26 had been proved to be sensitive to drought in many reports [60–62]. In the preparatory test, 3% PEG were found to inhibit seed germination, but soaking seed with melatonin can significantly promote soybean seed germination, so the germination rate is high. The germination potential of soybean seeds was determined after 7 days of germination, and the treatment of soaking seed with melatonin under 3% PEG was basically no different from CK, which indicated that melatonin soaking enhanced the resistance of seeds to water stress.

**Table 1:** Effect of soaking with melatonin on germination of soybean seeds under PEG stress.

Treatments		Germination rate/%	Germination potential/%	Germination index
**0**	**CK**	96.67±5.77a	96.67±5.77a	9.67±0.58a
	**M1**	90.00±10.0ab	96.67±5.77a	9.33±0.58a
	**M2**	93.33±5.77a	96.67±5.77a	9.50±0.50a
**3%**	**CK**	73.33±11.55cd	80.0±10.0bc	7.67±1.04bc
	**M1**	93.33±5.77a	96.67±5.77a	9.50±0.50a
	**M2**	96.67±5.77a	96.67±5.77a	9.67±0.58a
**6%**	**CK**	63.33±11.55d	73.33±5.77c	6.83±0.76c
	**M1**	76.67±11.55bcd	83.33±5.77bc	8.00±0.87bc
	**M2**	83.33±5.77abc	90.00±0.00ab	8.67±0.29ab
***CV* (%)**		13.85	9.97	11.81

Each value indicated is the means of three replicates recorded ± S.E. Means followed by the same letters are not significantly different at *P*≤0.05 within the same column. CV: Coefficient of variation.

**Table 2:** Effect of soaking with melatonin on radicle shape and biomass of soybean seeds under PEG stress.

Treatments		Root length /cm	Root surface area /cm^2^	Root volume /cm^3^	Fresh weight /g	Dry weight /g
**0**	**CK**	13.43±0.02e	6.09±0.01e	0.25±0.002bc	0.663±0.004a	0.043±0.002b
	**M1**	13.70±0.02c	6.26±0.01d	0.29±0.002ab	0.496±0.008c	0.047±0.002a
	**M2**	14.83±0.04a	7.47±0.01b	0.31±0.002a	0.523±0.002b	0.045±0.002b
**3%**	**CK**	12.08±0.09g	5.84±0.01g	0.25±0.001bc	0.460±0.002d	0.030±0.001de
	**M1**	12.51±0.03f	6.03±0.01f	0.28±0.002ab	0.407±0.003g	0.029±0.002e
	**M2**	14.40±0.02b	7.75±0.01a	0.27±0.003bc	0.433±0.002f	0.042±0.001b
**6%**	**CK**	12.08±0.10g	5.67±0.02h	0.23±0.002c	0.454±0.003e	0.033±0.002c
	**M1**	12.53±0.01f	5.07±0.03i	0.27±0.061b	0.364±0.003h	0.032±0.002d
	**M2**	13.58±0.01d	6.82±0.04c	0.25±0.001bc	0.412±0.001g	0.032±0.001cd
***CV* (%)**		6.49	7.54	13.67	19.00	18.63

Each value indicated is the means of three replicates recorded ± S.E. Means followed by the same letters are not significantly different at *P*≤0.05 within the same column. CV: Coefficient of variation.

Water stress damages the plant membrane system mainly via an imbalance in intracellular ROS (O_2_^·−^ and H_2_O_2_) production and clearance [63]. With the continuous accumulation of intracellular ROS, lipid membrane peroxidation gradually increases, and lipid membrane permeability and MDA contents continue to increase, resulting in damage to the membrane system and decreased cell organization [64]. In our study, the H_2_O_2_ content O_2_^·−^ production rate (Fig 2A and 2B) and EL (Fig 6A) in soybean increased significantly with increasing degree of PEG stress. However, after the seeds were soaked with melatonin, the content of H_2_O_2_ and the production rate of O_2_^·−^ reduced. These results are similar to those of Ding F [65] in tomato. Moreover, the MDA content and EL also significantly decreased. The reason may be that the decrease of H_2_O_2_ and O_2_^·−^ reduces the degree of membrane lipid damage, thus maintaining the health of the membrane system. This phenomenon can be seen from the results of the root tip staining and the cellular ultrastructural observations (Figs 3–5). Interestingly, we found that, after the seeds were soaked with melatonin, the content of H_2_O_2_ significantly decreased, and there was no significant difference in the production rate of O_2_^·−^ in the absence of water stress. In addition, under PEG stress, it can be seen from the histogram that soaking with 500 μmol·L^-1^ melatonin has a more significant effect on the reduction in H_2_O_2_ content, O_2_^·−^ production, EL and MDA content than that of 300 μmol·L^-1^ (Figs 2 and 6).

Water stress leads to the accumulation of ROS, which might damage to plants cells [66]. However, plants could remove ROS and maintain the balance of cellular oxidative metabolism under adverse conditions, this defense ability is mainly manifested in the increase of antioxidant enzyme activity, which is an inherent property of the plant [67]. However, the accumulation of excess ROS impaired the cell balance under water stress [68]. Therefore, plant defensive capabilities could be enhanced by controlling and removing excess ROS [69]. Protective enzymes such as SOD, POD, CAT and APX [70] removed and transformed O_2_^·−^, H_2_O_2_ and other ROS to gradually relieve overoxidation of cells. SOD could dismutase O_2_^·−^ into H_2_O_2_ to reduce cell damage, POD could use the different substrates as electron donors to restore H_2_O_2_ into water, APX could use ascorbic acid as an electron donors to restore H_2_O_2_ into water, and CAT could break H_2_O_2_ into water and oxygen [53]. In our study, the activity of POD, CAT and APX increased with increasing PEG stress (Fig 7B–7D), and the results showed that soybean radicles responded to water stress caused by PEG. SOD activity first increased but then decreased (Fig 7A). This phenomenon may have occurred with an increase in stress levels, excessive ROS restrict the activity of SOD, reducing its activity. The activity of POD, CAT and APX further increased after the soybean seeds were soaked in melatonin (Fig 7B–7D). Among the activity of these enzymes, under 6% PEG stress, the activity of SOD in the M1 treatment was significantly greater than that in the CK treatment (Fig 7A). Regardless of the presence of 3% or 6% PEG, the activity of POD and APX significantly increased in the M2 treatment (Fig 7B and 7D). The regulation of the various enzymes activity in different melatonin concentrations was inconsistent under different water stress conditions. This finding may be due to the different characteristics of each antioxidant enzyme. According to the report of Foyer [71], an increase in a single antioxidant enzyme is not enough to increase the stress response of plants; the response must be achieved by the synergy among various protective enzymes. The interaction of these four enzymes may thus play a role in balancing oxidative metabolism in plants to reduce oxidative damage [72, 73].

Proline and soluble proteins are two common osmotic adjustment substances present in plants under water stress [74]. An increase in these proteins helps to protect the integrity of cells by reducing moisture loss under water stress [75]. In our study, the contents of proline and soluble proteins in soybean radicles increased under PEG stress (Fig 8A and 8B). These results indicated that the seed soaking with melatonin promoted the synthesis of organic solutes in soybean cells, increased the content of osmotic regulators, reduced cell osmotic potential, maintained osmotic regulatory system of soybean, ensured the integrity of cell membrane structure, improved the adaptability of soybean plants to drought stress. The application of exogenous melatonin (300 and 500 μmol·L^-1^) under water stress (3% and 6% PEG), compared with CK, the proline and soluble protein contents were significantly increased, especially 500 μmol·L^-1^ melatonin. Similar results were reported by Khan [76] and Meng [45].

The GGE biplot (Fig 9) showed that soaking with melatonin can promote the germination of soybean seeds and can effectively alleviate the inhibitory effects of PEG stress on the germination of soybean seeds. Based on the results of this study, the transcriptome analysis or other molecular biology methods of soybean radicles would be carried out in order to explore the metabolic pathways and differential functional genes of melatonin soaking at soybean germination stage. And finally, the mechanism of melatonin soaking to alleviate drought stress injury was clarified.

## Conclusion

With increasing PEG stress, the content of H_2_O_2_, O_2_^·−^, EL and MDA increased significantly, resulting in cell membrane lipid damage, enhanced the permeability of the cell membrane, cellular osmotic potential and oxidative metabolism imbalance, and finally inhibiting the germination of soybean seeds (germination parameters, root length, root volume, root surface area and biomass decreased). Among the degrees of 3% and 6% PEG stress, 6% PEG stress had the greatest impact on the germination of soybean seeds. After being subjected to PEG stress, soybean seeds soaked with melatonin can induce more antioxidants (SOD, POD, CAT and APX), which can able to remove excess ROS. Moreover, the increase in osmotic adjustment substances (soluble proteins and proline) can improve the balance of cellular osmotic potential, reduce cell water loss, slow cell damage, improve the germination parameters of seeds and promote the growth of radicles. The negative effects of PEG stress on seed germination were ultimately alleviated. The regulatory effect of 500 μmol·L^-1^ melatonin was better than that of 300 μmol·L^-1^ melatonin. This research provides valuable foundation for enhancing the germination of soybean seeds tolerance and adaptation to drought stress, and the underlying regulatory mechanism needs to be studied further in the future.

## Supporting information

S1 Data (XLSX)
